# Maternal Obesity Enhances Collagen Accumulation and Cross-Linking in Skeletal Muscle of Ovine Offspring

**DOI:** 10.1371/journal.pone.0031691

**Published:** 2012-02-14

**Authors:** Yan Huang, Jun-Xing Zhao, Xu Yan, Mei-Jun Zhu, Nathan M. Long, Richard J. McCormick, Stephen P. Ford, Peter W. Nathanielsz, Min Du

**Affiliations:** 1 Developmental Biology Group, Department of Animal Science, Center for the Study of Fetal Programming, University of Wyoming, Laramie, Wyoming, United States of America; 2 Center for Pregnancy and Newborn Research, Health Sciences Center, University of Texas, San Antonio, Texas, United States of America; 3 Department of Animal Sciences, Washington State University, Pullman, Washington, United States of America; University of Colorado Denver, United States of America

## Abstract

Maternal obesity (MO) has harmful effects on both fetal development and subsequent offspring health. We previously demonstrated that MO enhances collagen accumulation in fetal skeletal muscle, but its impact on mature offspring muscle collagen accumulation is unknown. Ewes were fed either a control diet (Con, fed 100% of NRC nutrient recommendations) or obesogenic diet (OB, fed 150% of NRC nutrient recommendations) from 60 days before conception to birth. All ewes received the Con diet during lactation. Male offspring were euthanized at 2.5 years (mean) and the left *Longissimus dorsi* (LD) muscle and semitendinosus (ST) muscle were sampled. Collagen concentration increased by 37.8±19.0% (*P*<0.05) in LD and 31.2±16.0% (*P*<0.05) in ST muscle of OB compared to Con offspring muscle. Mature collagen cross-linking (pyridinoline concentration) was increased for 22.3±7.4% and 36.3±9.9% (*P*<0.05) in LD and ST muscle of OB group respectively. Expression of lysyl oxidase, lysyl hydroxylase-2b (LH2b) and prolyl 4-hydroxylase (P4HA) was higher in OB LD and ST muscle. In addition, the expression of metalloproteinases (MMPs) was lower but tissue inhibitor of metalloproteinases (TIMPs) was higher in OB offspring muscle, indicating reduced collagen remodeling. MO enhanced collagen content and cross-linking in offspring muscle, which might be partially due to reduced collagen remodeling. Our observation that the collagen content and cross-linking are enhanced in MO offspring muscle is significant, because fibrosis is known to impair muscle functions and is a hallmark of muscle aging.

## Introduction

Obesity is an increasingly critical problem and impacts general health worldwide in epidemic proportions. According to a report from Trust for America's Health (2010), 38 states had adult obesity rates above 25%. It was predicted that, by 2015, 75% of adults will be overweight or obese, and 41% will be obese, and women who are 20–34 years old have the fastest increase rate of obesity and overweight [1]. Maternal obesity (MO) combined with high-energy diets is harmful to fetal development, predisposing offspring to hypertension, type II diabetes, dyslipidemia, and heart disease [2], [3], [4]. However, mechanisms linking MO to adverse physiological changes in offspring remain poorly defined.

We have developed a pregnant ewe model of MO and over-nutrition in which females become obese prior to conception and remain obese throughout pregnancy [5], [6]. Using this model, when offspring from obese mothers were subjected to a bout of *ad libitum* feeding as adults, they exhibited decreased insulin sensitivity and increased adiposity compared to offspring from control mothers fed only to requirements [7]. Because skeletal muscle is the main tissue responsible for insulin stimulated glucose and fatty acid utilization, it is likely that offspring skeletal muscle function was impaired by MO. Skeletal muscle fibrosis impairs muscle function, and increasing fibrosis and fat infiltration is a hallmark of aging [8]. Limited studies indicate that maternal nutrition affects fibrogenesis in fetal and offspring skeletal muscle. Maternal nutrient restriction in swine increases collagen content in offspring skeletal muscle [9]. Our earlier study in the fetuses of MO ewes revealed enhanced transforming growth factor β (TGF-β) signaling and collagen accumulation in fetal muscle associated with an inflammatory response in skeletal and cardiac muscle born to obese mothers [6], [10]. TGF-β stimulates fibrosis partially via decreased expression of matrix metalloproteinases (MMPs), a family of functionally related enzymes that cleave extracellular matrix (ECM) components, and increased expression of tissue inhibitor of metalloproteinases (TIMPs) which play an important role in regulating ECM turnover [11], [12]. Lysyl oxidases catalyze a key step in the cross-linking of collagen and elastin [13], critical for the proper function of connective tissue. To date, the impact of developmental programming of MO on collagen accumulation, cross-linking and remodeling in offspring muscle has not been tested. We hypothesized that effects shown in fetuses of MO ewes would persist into adult life. The results presented here show that MO induced accumulation and enhanced cross-linking, which should be due to the inhibited remodeling of collagen in offspring skeletal muscle.

## Results

### Maternal and offspring weights

OB ewes increased their body weight by 31% from diet initiation to mating (72.1±3.7 and 94.7±3.9 kg, respectively; *P*<0.05) and increased 47% and 54% in body weight from diet initiation to day 75 and day 135 of gestation, respectively (*P*<0.05). In contrast, Con ewes, whose body weight was similar (*P* = 0.57) to that of OB ewes at diet initiation (70.4±3.1 kg), exhibited only modest non-significant (*P*>0.24) increases in body weight from diet initiation to conception (2.7%), day 75 (7.1%) or day 135 (13.1%) of gestation. Similarly, body condition score of OB ewes increased (*P* = 0.02) from diet initiation to mating (5.0±0.2 and 7.1±0.3, respectively), and further increased (*P*<0.05) to 7.9±0.2 by day 75, and 8.7±0.2 by day 135. The body condition score of control ewes remained relatively constant (*P*>0.29) from diet initiation to day 135 of gestation (5.1±0.5).

At 19 weeks of age, no difference in offspring body weight was observed; but at necropsy following 12 weeks of *ad libitum* feeding, OB male offspring weighed slightly more than Con male offspring (113.2±3.0 vs. 103.7±3.0 kg). The weight of the left LD and left ST muscle was similar (*P*>0.10) between Con and OB male offspring (1,158±41 vs. 1,162±45 g for LD, and 285±11 vs. 280±14 g for ST muscle).

### Collagen content in OB and Con offspring muscle

**Figure 1:**
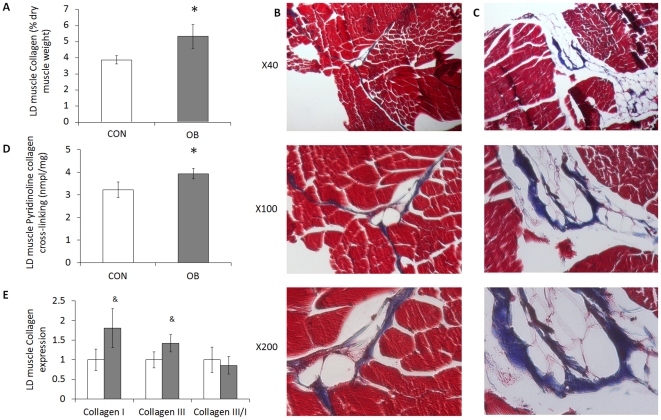
Collagen content, cross-linking, and mRNA expression in LD muscle of offspring sheep from Con (□) and OB (▪) ewes. *A*: Collagen content calculated based on the hydroxylproline concentration showing an increase in collagen concentration in OB offspring LD muscle (% dry muscle weight). *B* and *C*: Representative images of LD muscle from Con and OB offspring sheep at different magnifications. Transverse section of LD muscle stained with Masson's trichrome showing muscle fibers (red) and connective tissue (blue). *D*: Collagen cross-linking was higher in OB offspring muscle based on the concentration of pyridinoline (nmole/mg of dry muscle weight). *E*: Type I and III collagen expression and type III/I ratio of LD muscle. (*P<0.05, ^&^ *P*<0.10; Mean ± SE; n = 7).

**Figure 2:**
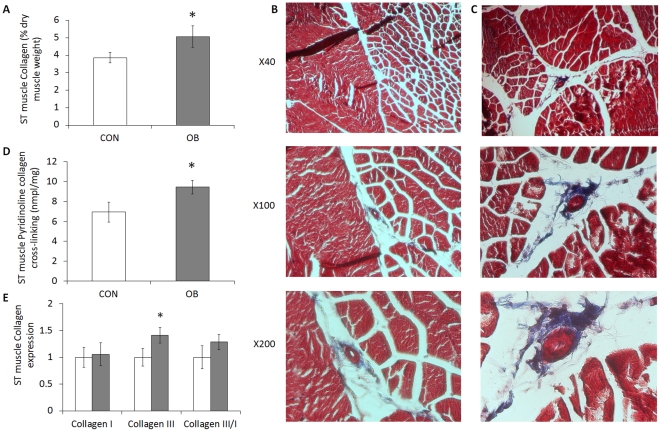
Collagen content, cross-linking, and mRNA expression in ST muscle of offspring sheep from Con (□) and OB (▪) ewes. *A*: Collagen content calculated based on the hydroxylproline concentration showing an increase in collagen concentration in OB offspring ST muscle (% dry muscle weight). *B* and *C*: Representative images of ST muscle from Con and OB offspring sheep at different magnifications. *D*: Collagen cross-linking was higher in OB offspring muscle based on the concentration of pyridinoline (nmole/mg of dry muscle weight. *E*: Type I and III collagen expression and type III/I ratio of ST muscle. (*P<0.05; Mean ± SE; n = 7).

Collagen content, as measured by hydroxyproline concentration, increased by 37.8±19.0% (*P*<0.05) in LD muscle of OB compared to Con offspring muscle (Fig. 1A). As shown by trichrome staining, more collagen was detected in OB LD muscle (Fig. 1B and 1C). The collagen content in ST muscle was also higher (31.2±16.0%, *P*<0.05) in OB muscle than in Con muscle (Fig. 2A), a result further confirmed by trichrome staining (Fig. 2B and 2C).

Pyridinoline is a trivalent, endpoint crosslink on the hydroxyllysine pathway. Pyridinoline content was also increased (22.3±7.4%, *P*<0.05; and 36.3±9.9%, *P*<0.05) in both LD and ST muscle of OB compared with Con offspring muscle (Fig. 1D and 2D).

### Collagen mRNA expression

The mRNA expression for both collagen I and collagen III in LD muscle tended to increase in OB compared to Con offspring (80.7±49.8%, *P*<0.10; and 42.2±21.6%, *P*<0.10, respectively) (Fig. 1E). In ST muscle, mRNA expression of collagen III was significantly increased (45.5±14.6%, P<0.05) in OB compared with Con offspring (Fig. 2E). There was no difference in collagen I/III ratio in LD or ST muscle.

### TGF-β signaling pathway and p38 phosphorylation

**Figure 3:**
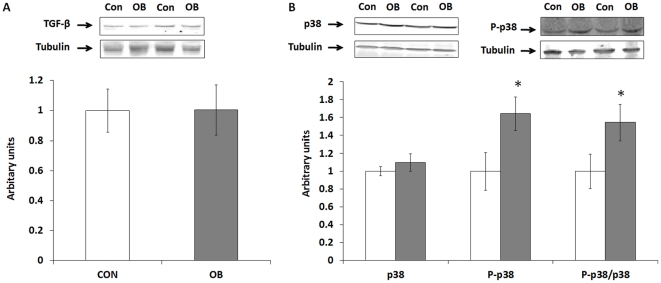
TGF-β, p38 and its phosphorylation in LD muscle of offspring sheep from Con (□) and OB (▪) ewes. Tubulin was used as a loading control. *A*: Western blotting showed no difference in TGF-β signaling in LD muscle. *B*: An increase in p38 phosphorylation was observed in OB compared to Con offspring LD muscle. (Arbitrary units; *P<0.05; Mean ± SE; n = 7).

LD and ST muscle TGF-β (Fig. 3A), Smad3, phospho-Smad3, and ST muscle p38 (data not shown) did not differ between Con and OB groups. Although no difference in total p38 protein concentration in LD muscle was observed between OB and Con fetal muscle, p38 phosphorylation was 64.3±18.9% higher (*P*<0.05) in Con compared to OB LD muscle (Fig. 3B). The phospho-p38/p38 ratio was also higher (54.5±20.2%, *P*<0.05) in OB compared with Con offspring muscle (Fig. 3B).

### mRNA expression of lysyl oxidase, LH2b, and P4HA

**Figure 4:**
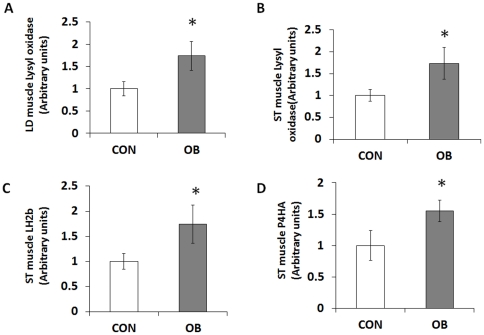
Relatvie lysyl oxidase, lysyl hydroxylase 2b (LH2b), and prolyl 4-hydroxylase α subunit (P4HA) mRNA expression in LD and ST muscle of offspring sheep from Con (□) and OB (▪) ewes. Enzymes expression increased in OB offspring group compared to Con offspring. (Arbitrary units; *P<0.05; Mean ± SE; n = 7).

Lysyl oxidase, LH2b, and P4HA are key enzymes regulating collagen biosynthesis and cross-linking. Expression of lysyl oxidase was higher in OB (74.0±32.3%, *P*<0.05; and 73.6±36.6%, *P*<0.05) for both LD and ST muscle (Fig. 4A and 4B). Expression of LH2b and P4HA was higher (74.2±38.2%, *P*<0.05; and 54.8±17.3%, *P*<0.05) in OB compared to Con ST muscle (Fig. 4C and 4D).

### mRNA expression of MMPs and TIMPs

**Figure 5:**
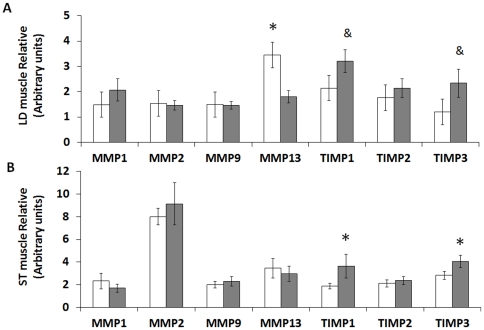
Matrix metalloproteinase (MMP) and tissue inhibitor of metalloproteinase (TIMP) expression in LD and ST muscle of offspring sheep from Con (□) and OB (▪) ewes. *A*: MMP13 was higher while TIMP1 and TIMP3 tended to be higher in OB compared with Con offspring LD muscle. *B*: TIMP1 and TIMP3 were higher in OB compared with Con offspring ST muscle. (Arbitrary units; *P<0.05, ^&^ *P*<0.10; Mean ± SE; n = 7).

MMPs and TIMPs are important regulators of collagen remodeling. LD muscle of Con offspring expressed more MMP13 (3.45±0.64 vs. 1.80±0.24, *P*<0.05) than OB offspring muscle. TIMP1 and TIMP3 tended to increase in OB compared with Con fetuses (2.15±0.45 vs. 3.21±0.45 arbitrary units, *P*<0.10; and 1.20±0.38 vs. 2.34±0.55 arbitrary units, *P*<0.10, respectively) (Fig. 5A). TIMP1 and TIMP3 mRNA expression was higher (3.64±1.05 vs. 1.89±0.25 arbitrary units, *P*<0.05; and 4.07±1.55 vs. 2.83±0.36 arbitrary units, *P*<0.05) in OB compared with Con ST muscle (Fig. 5B).

## Discussion

Recent evidence suggests that MO induces fetal and offspring obesity, metabolic abnormalities and inflammation [14], [15], [16], but the underlying mechanisms remain poorly defined. We have recently established an obese sheep model to study fetal development and developmental programming resulting from pre-pregnancy obesity and high energy diet in a paradigm that resembles the situation in obese women who become pregnant [17]. Because the ratio of fetal to maternal body mass and the single or twin pregnancies of sheep are very similar to those of humans, pregnant sheep have been widely used as an animal model to study developmental programming resulting from challenges similar to those experienced in human pregnancy [18], [19], [20], [21], [22], [23].

Skeletal muscle constitutes about 40–50% of body mass [24] and is the main peripheral tissue responsible for the oxidation of glucose and fatty acids [25]. The fetal period is crucial for skeletal muscle development, because no net increase in the number of muscle fibers occurs after birth [26], [27]. Fibrogenesis which forms endomysium and perimysium is actively ongoing during the fetal stage, and excessive fibrogenesis impairs muscle function [28]. Indeed, increased adiposity and fibrosis during aging is associated with a progressive loss of muscle mass [8], [28], resulting in a decline in muscle structural integrity and functional capacity [29]. Therefore, excessive fibrogenesis during fetal skeletal muscle development, if it persists into adult life, may have important negative physiological consequences for offspring health. Our previous studies demonstrated that MO induces changes in fetal skeletal muscle development, including an increase in intramuscular adipocytes and fibrosis [6], [12], changes not typically observed until later in life. Here, we further studied the long-term impact of MO on fibrosis of offspring muscle. Two different muscles were chosen for this study; LD muscle has low collagen content and is mainly composed of fast muscle fibers while ST muscle has high collagen content and a high ratio of slow muscle fibers.

Fibrosis is characterized by the accumulation of collagen inside tissues, and collagen homeostasis is maintained through a balance of synthesis and degradation. In our previous study, we demonstrated that collagen synthesis was enhanced in the skeletal muscle of OB compared to Con fetal sheep muscle, associated with enhanced TGF-β and p38 signaling in OB fetal muscle [6], [12]. MO induced inflammation in fetal skeletal muscle as demonstrated by the up-regulation of nuclear factor κ light-chain enhancer of activated B cells and Jun NH2-terminal kinase pathways [6], [25], changes that were accompanied by enhanced fibrosis [12]. Muscle inflammation induces expression of TGF-β and promotes connective tissue expansion during muscle regeneration, thus increasing collagen accumulation in muscle [30]. In short, enhanced collagen accumulation during the fetal stage appears to be mainly due to the increase in collagen synthesis [12].

Consistent with our results in fetal muscle, a higher collagen content was also detected in OB offspring muscle, which is similar to our previous observation of offspring of OB sheep [31]. However, no difference in TGF-β content was observed in this study. Despite the lack of different in TGF-β content, p38 activation was higher in MO offspring muscle. Because p38 signaling is essential for TGF-β down-stream signaling [32], [33], higher p38 activation in OB offspring muscle indicates that down-stream TGF-β signaling was more active in OB muscle compared to Con muscle. Because TGF-β signaling regulates collagen synthesis and fibrosis [30], [34], the higher p38 activation is expected to enhance down-stream TGF-β signaling, which might partially explain the enhanced collagen accumulation in offspring muscle of obese mothers. Our observation that the collagen content and cross-linking are enhanced in MO offspring muscle is significant, because it will likely to impair muscle functions. We previously observed that maternal obesity leads to insulin resistance in offspring lambs [35]. In previous studies, maternal obesogenic diet impaired muscle development and contraction force in offspring rats [36], [37], which are consistent with our observations.

Collagen type I and III are the most abundant collagen types in skeletal muscle [38]. These collagens are fibril forming and serve as a supportive structure in muscle tissue [39]. Collagen type I fibers are characterized by thick fibers while type III fibers are thin fibers with slightly different physical properties [40]. There is more type I than type III collagen in slow muscles, while the proportion of type III collagen is usually greater in fast muscles [41]. In this study, the offspring skeletal muscle collagen III/I ratio was not altered by MO, which might be associated with the relatively low animal number used in the current study. However, expression of both type I and III collagen had tendency to be higher in OB offspring LD muscle, and collagen III was increased in ST muscle of OB group.

To further explore mechanisms leading to enhanced collagen accumulation in offspring, we analyzed key enzymes regulating collagen remodeling. One of the major functions of MMPs is to catalyze collagen degradation and they have critical roles in skeletal muscle fibrosis, damage repair and connective tissue remodeling [42], [43], [44]. The activity of MMPs is inhibited by the TIMPs [45], [46] and the balance between MMP and TIMP is crucial for numerous physiological processes [45]. In the current study, we observed a decrease of MMP13 expression level in OB offspring LD muscle. Of course, though MMP mRNA expression is correlated with MMP protein content, it might not be necessarily correlated with the activity of MMPS, as MMPs are secreted intracellularly as latent, non-active enzymes. We also observed that TIMP1 and TIMP3 mRNA expression was higher in ST muscle of OB offspring. This increase of TIMP and TIMP3 might inhibit activities of MMPs in OB offspring skeletal muscle, inhibiting collagen degradation and remodeling. As a result, collagen accumulated in OB offspring muscle compared to Con muscle, leading to fibrosis. The mechanisms responsible for the changes in mRNA expression of MMPs and TIMPs are not clear, but persistent low grade inflammation and enhanced p38 signaling in offspring muscle may inhibit collagen remodeling and lead to fibrosis [47], [48]. In this regard we have previously reported inflammation in offspring muscle of OB sheep [31], and consistent with the enhanced p38 activation observed in OB offspring muscle of the current study.

Intermolecular crosslinking provides stability to collagen fibrils [49], [50], [51]. Crosslinking is initiated by oxidative deamination of selected telopeptide and helical collagen lysine residues, a critical step catalyzed by lysyl oxidase. Lysyl oxidase was more abundant in OB compared to Con offspring muscle, consistent with the enhanced collagen cross-linking detected in OB offspring muscle. The higher expression of lysyl oxidase likely results from inflammation and obesity in offspring. Inflammation induces lysyl oxidase expression via hypoxia-inducible factor 1α [52]. Obesity related syndromes, such as hypertension [53] and diabetes [54], are reported to be associated with an increased lysyl oxidase mediated collagen cross-linking. Thus, lysyl oxidase plays a key role in fibrotic pathogenesis [55].

In conclusion, our findings demonstrate that MO enhances muscle collagen accumulation, which might be partially due to the inhibition of remodeling in offspring muscle by reducing MMP13 and enhancing TIMP1 and TIMP3 expression. MO also promotes collagen cross-linking in offspring muscle, associated with enhanced lysyl oxidase expression. To our knowledge this is the first report that collagen accumulation and remodeling in offspring skeletal muscle is programmed by maternal nutrition and our findings are of importance in relation to the well established occurrence of insulin resistance and muscle weakness that accompanies fetal exposure to maternal obesity.

## Materials and Methods

### Ethics Statement

All animal procedures were approved by the University of Wyoming Animal Care and Use Committee (A-3216-01).

### Care and use of animals

Our previous animal model of [31] was broadly adapted for use here, with the following modifications: Multiparous Rambouillet/Columbia cross ewes (3–5 years of age with 2–4 previous pregnancies) were used to produce offspring for the current study. During each of 2 consecutive years, groups of ewes were mated with the same ram. Each year beginning 60 days before conception and continuing to the day of parturition, ewes were randomly assigned to be fed either a highly palatable diet at 100% of National Research Council (NRC) recommendations for energy (Con) [56] or 150% of NRC's recommended energy requirements (OB). All ewes were weighed at weekly intervals, and rations were adjusted for weekly changes in metabolic body weight (BW^0.75^) [57]. Body condition was scored at monthly intervals to evaluate changes in fatness. A body condition score of 1 (emaciated) to 9 (obese) was assigned by two trained observers after palpation of the transverse and vertical processes of the lumbar vertebrae (L2 through L5) and the region around the tail head [58].

During lactation, ewes were fed a diet meeting 100% of NRC nutrient requirements. After weaning, male lambs were fed at NRC recommendations for growing lambs and to maintenance as adults. Prior to two weeks of age, lambs were tail-docked and males were castrated as per Federation of Animal Science Societies recommendations [59]. Four 3 year-old male offspring (from year 1 of the trial), and three 2 year-old male offspring (from year 2 of the trial) born to Con (n = 7) and OB (n = 7) ewes respectively were placed on a 12 week *ad libitum* feeding trial as previously described in order to measure voluntary feed intake [7]. At the end of the feeding trial, male offspring were weighed, and euthanized with an overdose of sodium pentobarbital (Beuthanasia-D Special; Schering-Plough Animal Health, Union, NJ). The left *Longissimus dorsi* (LD) muscle was sampled over the 13^th^ rib, immediately after euthanization and weighed. Surface tissues were trimmed; one piece of muscle was sampled at the anatomic center of the muscle and snap-frozen in liquid nitrogen for biological analyses, and another piece was fixed in fresh paraformaldehyde before being embedded in paraffin. The remaining left LD was dissected and weighed, and its weight was added to the sample weights to calculate total LD weight. The *Semitendinosus* (St) muscle was sampled and weighed similarly.

### Histochemical analyses

Muscle samples were fixed in 4% (wt/vol) paraformaldehyde in phosphate buffer (0.12 M, pH 7.4), embedded in paraffin, and sectioned at 10 µm. Sections were rehydrated by a series of incubations in xylene and ethanol solutions and then used for Masson Trichrome staining [60], which stains muscle fibers red, nuclei black, and collagen blue [10].

### Antibodies and Western Blot anaylsis

Antibodies against tubulin (no. 2128), TGF-β (no. 3711), Smad2/3 (no. 3102), phospho-Smad2/3 at Ser^423/425^ (no. 9520), p38 (no. 9212), and phospho-p38 at Thr^180/182^ (no. 9211) were purchased from Cell Signaling (Danvers, MA).

Muscle samples were washed with PBS and lysed in a buffer containing 50 mM HEPES (pH 7.4), 2% SDS, 1% NP-40, 10% glycerol, 2 mM phenylmethylsulfonyl fluoride, 10 mM sodium pyrophosphate, 10 mg/ml aprotinin, 10 mg/ml leupeptin, 2 mM Na_3_VO_4_, and 100 mM NaF. Soluble proteins were recovered after a 10-min centrifugation (10,000× g), and their concentrations were determined according to the Bradford method (Bio-Rad Laboratories, Hercules, CA) [61]. Proteins in cell lysates were separated by SDS-PAGE and transferred to a nitrocellulose membrane. Membranes were incubated in a blocking solution with 1∶1 Odyssey Blocking Buffer (LI-COR Biosciences, Lincoln, NE) and PBS for 1 h. Membranes were incubated overnight in a 1∶1,000 to 1∶500 dilution of primary antibodies and a 1∶2,000 dilution of tubulin in 1∶1 Odyssey Blocking Buffer and PBS/T. Membranes were then incubated with IRDye 800CW Goat Anti-Rabbit Secondary Antibody or IRDye 680 Goat Anti-Mouse Secondary Antibody from LI-COR Biosciences (Lincoln, NE) at a 1∶10,000 dilution for 1 h in 1∶1 Odyssey Blocking Buffer and PBS/T with gentle agitation protecting from light. Membranes were visualized by an Odyssey Infrared Imaging System (LI-COR Biosciences). Density of bands was quantified and then normalized according to the tubulin content.

### Collagen concentration and pyridinoline cross-linking analyses

Muscle samples (0.1 g) were ground and dried in a convection oven at 60°C, and samples were weighed and hydrolyzed in 6 N HCl at 105°C for 16 h. An aliquot was removed for hydroxyproline determination, as described previously [62]. Collagen concentration (mg/g dry muscle weight) was calculated assuming collagen weighs 7.25 times of the measured weight of hydroxyproline [63].

After HCl digestion, the hydrolyzate was neutralized with NaOH and pyridinoline concentration was measured with Metra Serum PYD EIA kits (Quidel, San Diego, CA) following the company's protocol [64]. The pyridinoline concentration was expressed as nmole/mg of dry muscle weight.

### Real-time quantitative PCR (RT-PCR)

Total mRNA was extracted from muscle using TRI reagent (Sigma, St. Louis, MO) and reverse transcribed into cDNA by using a kit (Qiagen, Valencia, CA). RT-PCR was performed using an iQ5 RT-PCR detection system (Bio-Rad Laboratories, Hercules, CA). A SYBR Green RT-PCR kit from Bio-Rad Laboratories (Hercules, CA) was used together with the *Ovis aries* primers listed in Table 1. Each reaction yielded amplicons between 80 and 200 bp. PCR conditions were as follows: 20 s at 95°C, 20 s at 56°C, and 20 s at 72°C for 35 cycles. After amplification, a melting curve (0.01 C/s) was used to confirm product purity, and the PCR products were electrophoresed to confirm the targeted sizes. Results are expressed relative to tubulin.

**Table 1:** List of primers.

Primers	Forward sequence	Reverse sequence
collagen type I	5′-GGTGACAGGAAGTCCCAGAA-3′	5′-CTGTAGGTGAAGCGGCTGTT-3′
collagen type III	5′-GGTCAGCCTGGCGTCATGGG-3′	5′-GACCTCCAGGGCCACCTCGT-3′
Lysyl hydroxylase 2b	5′-ATGCCAATCAGGAGGATCTG-3′	5′-CAGGTAGCGTTTCCCAATGT-3′
Lysyl oxidase	5′-AGCTCAGCATACAGGGGAGA-3′	5′-CATCCATGCTGTGGTAATGC-3′
Prolyl 4-hydroxylase α	5′-GATAAGGCGCTTTTGCTCAC-3′	5′-ATCCACAGCAGCACCTTTCT-3′
MMP1	5′-ACTTGTACCGGGTGGCAGCG-3′	5′-GTTTGGGGGCCGACTGGCTG-3′
MMP2	5′-TGGCCATGCAATGGGGCTGG-3′	5′-TCAGGAGTGACGGGGCCGAG-3′
MMP9	5′-CGACGGCATGCTCTGGTGCA-3′	5′-CGCATTGCCGTCCTGGGTGT-3′
MMP13	5′-ACGCCGGACAAATGTGACCCTT-3′	5′-GCTGAGGATGCAGCCGCCAG-3′
TIMP1	5′-CCCAGCGCCCAGAGAGGCTA-3′	5′-TCTGTGGGTGGGGTGGGACG-3′
TIMP2	5′-CCCGGACGAGTGCCTCTGGA-3′	5′-CGCAGGAGCCGTCGCTTCTC-3′
TIMP3	5′-CCTCTCCCAGCGCAAGGGGT-3′	5′-GCCACCCTTCTGCCGGATGC-3′
Tubulin	5′-CGAGAGCTGTGACTGTCTGC-3′	5′-GGCATGACGCTAAAGGTGTT-3′

### Statistical analyses

Each animal was considered an experimental unit. Data were analyzed as a complete randomized design using general linear model (GLM) of SAS. No difference was observed in data from two age groups and, thus, these data were combined. Differences in mean values were compared by Tukey's multiple comparison test, and means ± SE was reported. Statistical significance was considered as *P*<0.05.
